# Assessment of Risk Factors for Atrial Fibrillation With a Particular Focus on Echocardiographic Parameters, in Patients With Acute Myocardial Infarction

**DOI:** 10.1002/clc.70114

**Published:** 2025-03-28

**Authors:** Beata Jacuś, Anna Milewska, Paweł Miękus, Marcin Konarzewski, Ludmiła Daniłowicz‐Szymanowicz, Andrzej Lubiński, Grzegorz Grześk

**Affiliations:** ^1^ Medical University of Gdańsk Nicolaus Copernicus University in Toruń Gdańsk Poland; ^2^ Cardiology and Internal Medicine Department University Center for Maritime and Tropical Medicine in Gdynia Gdynia Poland; ^3^ Department of Occupational Metabolic and Internal Diseases, Medical University of Gdańsk Gdańsk Poland; ^4^ Department of Cardiology and Clinical Pharmacology, Faculty of Health Sciences Ludwik Rydygier Collegium Medicum in Bydgoszcz, Nicolaus Copernicus University in Toruń Bydgoszcz Poland; ^5^ Cardiology Department St Vincent de Paul Hospital in Gdynia Gdynia Poland; ^6^ Department of Cardiology and Electrotheraphy Medical University of Gdańsk Gdańsk Poland

**Keywords:** acute myocardial infarction, atrial fibrillation, left atrium stiffness index, peak atrial longitudinal strain, speckle tracking echocardiography

## Abstract

**Background:**

Atrial fibrillation is the most common arrhythmia worldwide, affecting between 2% and 4% of population. The projected further progression is a reason to consider AF as a global epidemic problem. The efficiency in diagnosing new cases is still unsatisfactory.

**Methods:**

The prospective study included 74 patients hospitalized for acute myocardial infarction. Echocardiography with advanced assessment of the left atrium was performed on all patients. R Statistical Software was used for statistical and graphical processing.

**Results:**

Atrial fibrillation was first diagnosed in 13.5% of patients with acute myocardial infarction, and in 5.4% of the patients the diagnosis was made during the long‐term follow‐up period. Analysis of the data collected showed that patients with arrythmia were older (71.79 vs 63.5 years; *p* = 0.047), had a higher BMI (30.15 vs 26.76 kg/m^2^; *p* = 0.039) and had a higher CHA_2_DS_2_ VASc score (4.14 vs 3.02 points). Among the echocardiographic parameters, those that significantly differentiated patients with arrythmia included larger LA area (21.62 vs 18.84 cm^2^; *p* = 0.027), lower LAEF 4CH (43.46 vs 55.93%; *p* = 0.029), lower LAEF mean (44.08 vs 55.63%; *p* = 0.014), lower EI (1.03 vs 1.49; *p* = 0.032), lower LASr 4CH (19.08 vs 26.72%; *p* = 0.020), lower LASr mean (18.62 vs 26.73%; *p* = 0.009), higher E/e’ (12.62 vs 9.58; *p* = 0.01), higher LASI (0.95 vs 0.45; *p* = 0.016).

**Conclusions:**

Among the echocardiographic parameters, those that may indicate an increased risk of atrial fibrillation and could be implemented in clinical practice are LASr and LASI. Determining them in risk profiling and the implementation of individualized arrhythmia detection methods could increase diagnostic efficiency.

## Graphical abstract

The efficiency in diagnosing new cases of atrial fibrillation is still unsatisfactory. The diagnosis in a patient with coronary artery disease has a direct impact on the modification of pharmacotherapy. Among the echocardiographic parameters, one can distinguish those that may indicate an increased risk of atrial fibrillation.

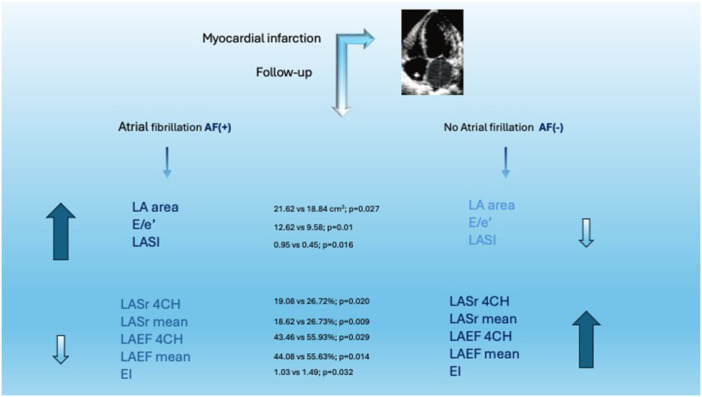

**Abbreviations**
AF: Atrial Fibrillation
BMI (kg/m^2^): Body Mass Index
CHARGE‐AF: Cohorts for Heart and Aging Research in Genomic Epidemiology Atrial Fibrillation
CHARGE‐AF adv: Cohorts for Heart and Aging Research in Genomic Epidemiology Atrial Fibrillation – advanced model
CHARGE‐AF sim: Cohorts for Heart and Aging Research in Genomic Epidemiology Atrial Fibrillation – basic model
CKD: Chronic Kidney Disease
DD: Diastolic Dysfunction
DM: Diabetes
DT (ms): Deceleration Time
EI: Expansion Index
ICM: Implantable Cardiac Monitor
LAEF 4CH (%): Left Atrium total Emptying Fraction in apical 4 CHamber View
LAEF mean (%): Letf Atrium total Emptying Fraction, mean value (in apical 4 and 2 CHamber view)
LAFI: Left Atrium Functional Index
LAScd: Left Atrial conduit Strain
LASct: Left Atrial contraction Strain
LASI: Left Atrium Siffness Index
LASr 4CH (%): Left Atrial reservoir Strain in apical 4 Chamber view
LASr mean (%): Left Atrial reservoir Strain, mean value (in apical 4 and 2 Chamber view)
LAVi: Left Atrium Volume index
LA area (cm^2^): Left Atrium area
LA max: Left Atrium maximal volume
LA min: Left Atrium minimum volume
LDL: Low Density Lipoprotein
LVEDd: Left Ventricle End Diastolic Diameter
LVOT VTI: Left Ventricular Outflow Track Velocity Time Integral
LV GLS (%): Left Ventricle Global Longitudinal Strain
NDF: Normal Diastolic Function
PALS (%): Peak Atrium Longitudinal Strain
ROI: Region of Interest
STE: Speckle Tracking Echocardiography
TCH: Total Cholesterol

## 1 Introduction

Atrial fibrillation is an arrhythmia presenting with rapid, uncoordinated electrical activation of the atria leading to ineffective contraction. According to current guidelines, an arrhythmia lasting at least 30 s recorded on a single‐lead electrocardiogram or an abnormal heart rhythm recorded on a 12‐lead electrocardiogram is required to make the diagnosis [1]. Worldwide, this type of arrhythmia is the most common and it affects between 2% and 4% of the adult population [2, 3, 4]. The projected further progression is a reason to look at atrial fibrillation as a global epidemic problem [5]. To date, many factors that may contribute to the development of arrhythmia have been described [6, 7, 8, 9, 10, 11]. The risk profile of arrhythmia varies, and it is extremely difficult to narrow down the most susceptible group of patients. This is one of the reasons for the still unsatisfactory success rate in diagnosing new cases of arrhythmia and thus the inability to implement preventive interventions and avoid complications. Significantly, the silent clinical course may affect up to 40% of the Atrial Fibrillation population and contribute to prolonging the time from the onset to diagnosis and implementation of therapy. No difference in all‐cause mortality, cardiovascular mortality or thromboembolic complications, including strokes, has been demonstrated when compared with full‐blown arrhythmia [12]. Atrial fibrillation is the most common arrhythmia, while coronary artery disease ranks first in prevalence among cardiovascular diseases in the human population [13, 14]. Both diseases share many risk factors [15]. The diagnosis of atrial fibrillation in a patient with coronary artery disease, including myocardial infarction, has a direct impact on modifying pharmacotherapy by increasing the CHA_2_DS_2_ VAS_c_ score, resulting in significant changes in antithrombotic theraphy [16, 17]. In summary, the consequences of an atrial fibrillation diagnosis in that population are significant. The increasing number of patients with arrhythmia, the high proportion of asymptomatic form, the possibility of rapid implementation of therapy, and the increasing variety of devices to facilitate diagnosis have strengthened the international initiative to implement screening methods for atrial fibrillation in everyday medical practice [18]. The main aim of this study was to assess risk factors for atrial fibrillation in patients with acute myocardial infarction. Clinical and laboratory parameters were thoroughly analyzed, and the assessment covered echocardiography in particular. The premise of the study was to identify those parameters that could be risk factors for arrhythmia.

## 2 Materials and Methods

### 2.1 Study Group

The prospective study included 74 patients hospitalized for acute myocardial infarction in the cardiology department of a district hospital. The recruitment period lasted 11 months. Inclusion criteria were a diagnosis of myocardial infarction, age over 18 years, and anticipated satisfactory cooperation during follow‐up period. Exlusion criteria were lack of informed consent and in‐hospital isolation due to SARS‐CoV‐2 infection. Subject were classified into four subgroups:

A: patients who completed the study without a diagnosis of arrhythmia; atrial fibrillation was not present in the history before hospitalization for myocardial infarction, did not occur during the index hospitalization and no arrhythmia was recorded in the longterm follow‐up

B: patients with a history of arrhythmia before hospitalization for myocardial infarction

C: patients whose arrhythmia was first diagnosed during the index hospitalization

D: patients in whom the arrhythmia was first diagnosed during long‐term follow‐up

Due to the small number of atrial fibrillation (AF) first diagnosed during the long term follow‐up, the population was finally divided into two gropus:

AF (−): patients who were not diagnosed with atrial fibrillation during the study

AF (+): patients in whom atrial fibrillation was first diagnosed during hospitalization for acute myocardial infarction or during the long‐term follow‐up

Patients with a medical history of atrial fibrillation present before the index hospitalization were excluded from the comparative statistical analysis. Finally, advanced calculations were performed on 72 patients.

Baseline characteristics are shown in Supplementary material (Tables S1–S4).

### 2.2 Clinical Evaluation

Using available clinical data, patients were assessed using the CHARGE‐AF (Cohorts for Heart and Aging Research in Genomic Epidemiology Atrial Fibrillation) and CHA_2_DS_2_ VASc scale. When estimating the risk of atrial fibrillation using the CHARGE‐AF calculator, the study employed both its basic model (CHARGE‐AF sim) and its extended model (CHARGE‐AF adv).

### 2.3 Echocardiography

Echocardiography was performed on equipment from General Electric Healthcare, Vivid S70N and Vivid E95. Analysis was performed using EchoPAC software, version 204, GE Vingmed Ultrasound AS, Horten, Norway. Detailed echocardiographic evaluation was performed for the following parameters: left atrial anteroposterior dimension, left atrium areas, left atrium volume indexed by body surface area, left atrium minimum and maximum volumes, as well as before the period of its contraction, left atrial strain in reservoir phase (LASr, left atrial reservoir strain), in the condiut phase (LAScd, left atrial conduit strain), in its active contraction phase (LASct, left atrial contraction strain), early diastolic flow maximum velocity (E wave), late distolic flow maximum velocity (A wave), early diastolic flow deceleration time (DT), maximum mitral annular velocities in the lateral and septal parts (e’), left ventricular end‐diastolic diameter, left ventricular systolic diameter, interventricular septal thickness, left ventricular posterior wall thickness, left ventricular ejection fraction in two‐dimensional imaging, left ventricular global longitudinal strain in two‐dimensional imaging. During the standard echocardiogram, the recommendations of the American Society of Echocardiography and the European Association for Cardiovascular Imaging were respected [19]. When using the acoustic marker tracking technique, the guidelines contained in the documents developed by the Working Group of the European Association for Cardiovascular Imaging and the American Society of Echocardiography were followed [20, 21] Using volumetric methods to assess the function of the left atrium, its global condition was expresses by the total emptying fraction (LAEF, Left Atrium Ejection Fraction), calculated from the formula: LAEF = [(LAmax‐LAmin)/LAmax]x100%, in which LAmax denoted the maximum volume of the left atrium and LAmin expressed its minimum volume. Left Atrium Functional Index (LAFI) was estimated using the equation: LAFI=LAEFxLVOT VTI/LAVi, where LAEF is the total Left Atrium Ejection Fraction, LVOT VTI is the Left Ventricular Outflow Track Velocity Time Integral, and LAVi is the indexed maximum Left Atrium Volume index. For volume assessment, the semi‐automatic algorithm included in the EchoPAC software was used. It was also used to assess left atrial strain using modern echocardiographic techniques (STE, Speckle Tracking Echocardiography). Individualized algorithm fo the left atrium (LA AFI) was used. The measurement zone within the left atrium was determined automatically, with manual adjustments to Region Of Interest (ROI), where necessary. The apical four‐chamber and two‐chamber projections were used. Left Atrium Stiffness Index (LASI) was calculated from the formula: LASI = E/e’:LASr, where E is the maximum velocity of early diastolic inflow into the left ventricle, e’ is the maximum velocity of mitral annulus motion, and LASr indicates left atrial strain during reservoir phase (the LASr value obtained in the apical four‐chamber view was used to calculate LASI). Three apical views were used for the measurement of LV GLS (Left Ventricle Global Longitudinal Strain). To determine peak atrial longitudinal strain (PALS) and LV GLS together, their absolute values were summed. The ventricular end‐diastolic period was taken as the initial measurement point in the assessment of left atrial strain, which means that the LASr values reported in the paper are the same as PALS.

### 2.4 Outcome and Follow‐Up

Partcipants were followed up for a period of 3 to 14 months. The average follow‐up period was 10.43 months (SD = 2.03). During the periodic assessment, electrocardiogram was performed and an interview was conducted, with particular attention to the presence of arrhythmia symptoms. Telephone consultations were conducted when there were difficulties due to restrictions related to the SARS‐Cov‐2 pandemic. The follow‐up method reflected current clinical practice.

### 2.5 Statistical Analysis

The data collected was entered into an electronic database and statistical analysis was carried out. R Statistical Software version 4.0.4 was used for statistical and graphical processing of the data [22]. Comparisons in terms of quantitative variables between the two groups were carried out by performing a series of Mann‐Whitney U test. The difference effect strength was calculated using Glass' biserial correlation coefficient. The choice of the nonparametric test was due to low numbers in one of the subgroups. The decision to merge groups of patients with arrhythmias diagnosed during hospitalization and during follow‐up was dictated by maximizing subgroup size and theoretical rationale. An analogous analysis of qualitative data was performed using the chi‐square test of independence. Pearson correlation analysis was used to determine the relationships between variables. A *p*‐value < 0.05 was considered statistically significant.

### 2.6 Approval of the Bioethics Committee

The study was conducted according to the ethical guidelines of the Declaration of Helsinki and approved by the Bioethics Committee at the Regional Chamber of Physicians in Gdańsk (KB 29‐20).

## 3 Results

The study included *N* = 74 patients with a diagnosis of acute myocardial infarction. Ultimately, statistical analysis of the data was performed in *N* = 72 patients. Women accounted for 26% and men for 74% of the population. The age of the average subject was approximately 65 years (M = 65.47; SD = 12.25). The BMI of the average subject averaged 27.42 kg/m^2^ (SD = 4.22). The maximum follow‐up time in the study was 14 months and the minimum was 3 months, with an average of 10.43 months (SD = 2.03). Atrial fibrillation was first diagnosed in acute myocardial infarction in 13.5% of patients, and in 5.4% the diagnosis was made during the long‐term follow‐up.

### 3.1 Demographic Factors and Laboratory Parameters

**Figure 1:**
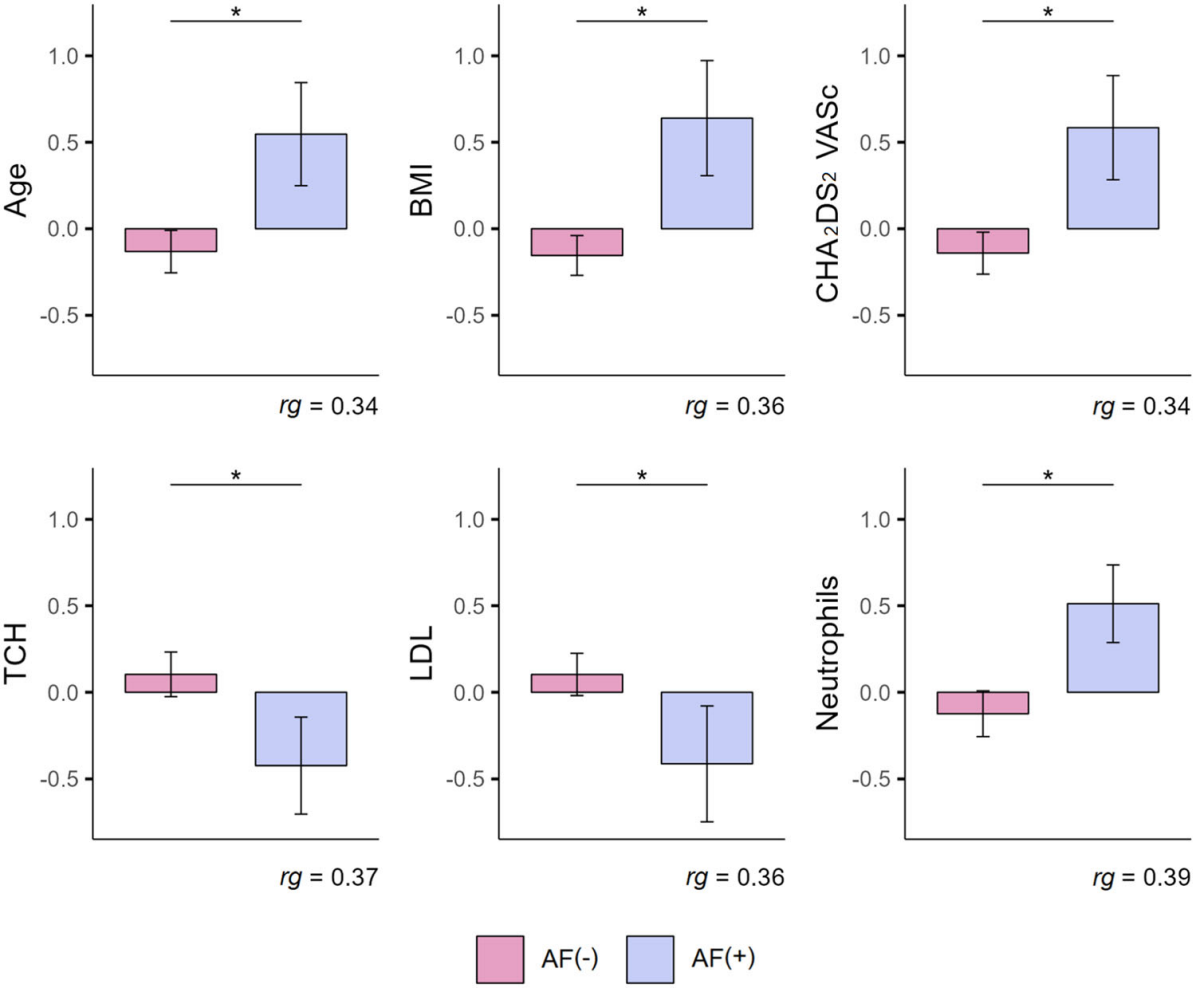
Comparison of groups of patients with a diagnosis of arrhythmia (atrial fibrillation) and without arrhythmia in terms of variables: Age, BMI, CHA2DS2VASc, TCH, LDL, Neutrophils. *Note:* Results are expressed in the standardized unit of measurement; Error whiskers represent the standard error of the mean. rg = Glass rank biserial correlation coefficient; *p < 0.05. **p < 0.01. ***p < 0.001. A, Age ≥ 65 to 74 years; A2, Age ≥ 75 years; BMI, Body Mass Index; CHA2DS2VASc‐ C, Congestive heart failure; D, Diabetes mellitus; H, Hypertension, LDL, low density lipoprotein cholesterol; S, prior Stroke or transient ischemic attack or thromboembolism; TCHL, total cholesterol; V, Vascular disease, Sex category.

Analysis of the data collected showed that patients with a diagnosis of arrhythmia were significantly older (*U* = 266; *p* = 0.047). The mean age of patient with a diagnosis of atrial fibrillation was 72 years (*M* = 71.79; SD = 13.61), while in the population without arrhythmia the mean age was 63.5 years (*M* = 63.5; SD = 11.39). It was found that BMI (Body Mass Index) differed significantly in the compared groups (*U* = 260.5; *p* = 0.039). A higher score was noted in patients with atrial fibrillation (*M* = 30.15 kg/m^2^; SD = 5.32) compared to subjects without arrhythmia (*M* = 26.76 kg/m^2^; SD = 3.75). Statistically significant differences in thromboembolic risk estimated using the CHA_2_DS_2_ VASc were confirmed (*U* = 266.5; *p* = 0.041). The group of subjects with arrhythmia had higher scores (*M* = 4.14; SD = 1.75) compared to the AF‐free group (*M* = 3.02; SD = 1.43). In terms of analysis of TCH (Total Cholesterol), there were significantly different results between the groups (*U* = 251.5; *p* = 0.034), with higher concentration among patients without arrhythmia (*M* = 176.48 mg/dl; SD = 43.2) compared to those with a diagnosis of atrial fibrillation (*M* = 153 mg/dl; SD = 46.68). It was found that the differences in LDL cholesterol levels were also statistically significant (*U* = 250.5; *p* = 0.038). Higher levels were found in the group without arrhythmia (*M* = 99.68 mg/dl; SD = 32.1) compared to subject with atrial fibrillation (*M* = 81.5 mg/dl; SD = 44.11). It was also shown that the percentage of Neutrophils was higher in patients withy arrhythmia (*M* = 76.94%; SD = 8.82) compared to the non‐arrhythmia group (*M* = 70.26%; SD = 10.53). The differences were statistically significant (*U* = 246; *p* = 0.023). The results obtained are shown in Figure 1.

### 3.2 Echocardiographic Parameters

**Figure 2:**
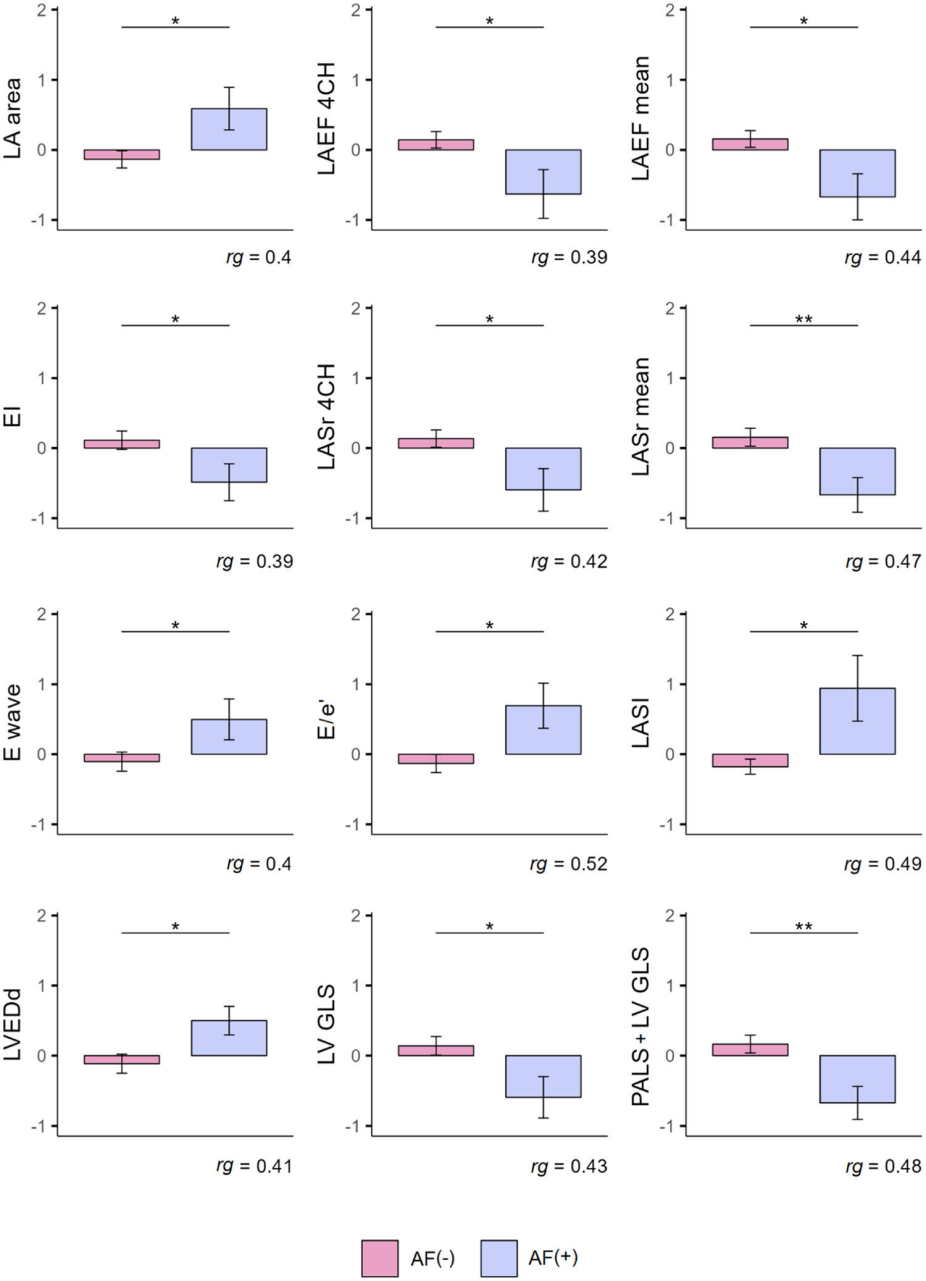
Comparison of the groups of patients with a diagnosis of arrhythmia (atrial fibrillation) and without arrhythmia in terms of variables: LA area (area of the left atrium), LAEF 4CH (total left atrium ejection fraction in apical four chamber position), LAEF mean (total left atrium ejection fraction, mean value), EI (Expansion Index), LAS 4CH (Left Atrial reservoir Strain in apical four chamber position), LASr mean (Left Atrial reservoir Strain, mean value), E wave, E/e’, LASI (Left Atrium Stiffness Index), LVEDd (Left Ventricle End Diastolic diameter), LVGLS (Left Ventricle Global Longitudinal Strain), PALS + LV GLS (Peak Atrial Longitudinal Strain + Left Ventricle Global Longitudinal Strain). Note: Results are expressed in the standardized unit of measurement; Error whiskers represent the standard error of the mean. rg = Glass’ biserial correlation statistic; *p < 0.05. **p < 0.01. ***p < 0.001.

The difference in LA area (Left Atrium area) between the gropus were significant (*U* = 223.5; *p* = 0.027). Patients with AF had a larger LA area (*M* = 21.62 cm^2^; SD = 4.2) compared to the patients in sinus rhythm (*M* = 18.84 cm^2^; SD = 3.59). According to the calculations, the differences between the groups in terms of LAEF 4CH (total Left Atrium Emptying Fraction in the apical 4 CHamber view) were statistically significant (*U* = 225.5; *p* = 0.029). Higher values were estimated in AF(−) patients (*M* = 55.93%; SD = 14.29) compared to AF(+) patients (*M* = 43.46%; SD = 20.22). It was shown that LAEF mean (total Left Atrium Emptying Fraction with the averaging of measurements from apical four and two‐chmber view) was higher in AF(−) subjects (*M* = 55.63%; SD = 12.52) and was lower in in AF(+) group (*M* = 44.08%; SD = 16.58); *U* = 203.5; *p* = 0.014. It was found that EI (Expansion Index) was higher in AF(−) patients (*M* = 1.49; SD = 0.76) than in patients with arrhythmia (*M* = 1.03; SD = 0.73); *U* = 223.5; *p* = 0.032. It was also proved that differences in LASr 4CH (Left Atrial reservoir Strain in apical 4 Chamber view) were significant (*U* = 216.5; *p* = 0.020). A higher strain characterized AF (−) population (*M* = 26.72%; SD = 9.77) compared to AF(+) patients (*M* = 19.08; SD = 11.42). It was found that LASr mean (Left Atrium reservoir Strain with averaging measurements from apical two and four‐chamber view) was higher in AF(−)group (*M* = 26.73%; SD = 9.54) compared to the arrhythmia group (*M* = 18.62%; SD = 8.79); *U* = 193.5; *p* = 0.009. E wave differed significantly (*U* = 172.5; *p* = 0.041). Higher velocities were calculated in patients with arrhythmia (*M* = 0.83 m/s; SD = 0.018) compared to the non‐arrhythmia group (*M* = 0.72 m/s; SD = 0.19). It was determined that the E/e’ variable was higher in AF(+) group (*M* = 12.62; SD= 3.76) compared to AF(−) group (*M* = 9.58; SD= 3.51); *U* = 128; *p* = 0.010. LASI was higher in AF(+) group (*M* = 0.95; SD = 0.66), and was lower in AF(−) patients (*M* = 0.45; SD = 0.35); *U* = 136; *p* = 0.016. LVEDd was greater in AF(+) subjects (M = 52.92 mm; SD = 3.73) than in AF(−) group (*M* = 49.79; SD = 5.22); U = 218.5; *p* = 0.022). LV GLS was higher in patients without AF (*M* = 14.49%; SD = 3.92) and lower in patients with arrhythmia (*M* = 11.47%; SD = 4.2); *U* = 174; *p* = 0.021. A higher score for the sum of PALS and LV GLS was observed for AF(−) subject (*M* = 39.92%; SD = 13.02) compared to the AF(+) group (*M* = 29.2%; SD = 10.54); *U* = 189.5; *p* = 0.007. The results obtained are shown in Figure 2.

### 3.3 Comorbidities

**Figure 3:**
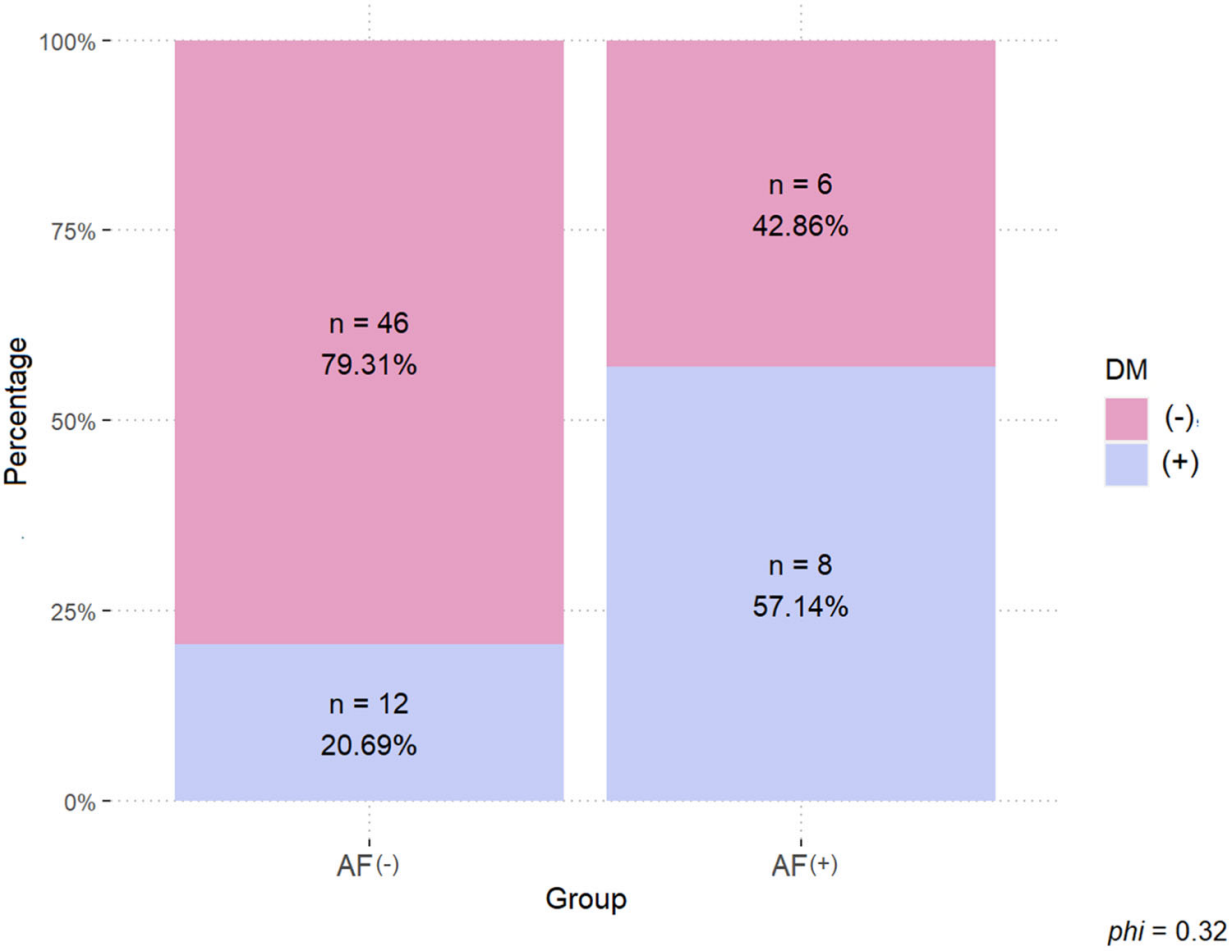
Comparison of groups of patients with a diagnosis of arrhythmia (atrial fibrillation) and without arrhythmia in terms of the prevalence of Diabetes Mellitus (DM). *Note:* phi = Yule coefficient; *p < 0.05. **p < 0.01. ***p < 0.001.

As a result of the analysis it was determined that there was a significant association between atrial fibrillation and DM (Diabetes); χ^2^(1) = 5.76; *p* = 0.016. Patients with AF had a higher pertcentage of DM (57.14%) than the AF(−) group. By analogy, in the non‐arrhythmia group, the percentage of those without DM was higher (79.31%) than in the AF(+) group (42.86%). The results obtained are shown in Figure 3.

### 3.4 Sex

It was not possible to perform a separate analysis for both sexes due to the small number of women with a diagnosis of atrial fibrillation in the study population (*n* = 4). It was decided to perform a comparative analysis in the male population in terms of 2 echocardiographic parameters which proved to be significant after studying the entire population and which have the potential to be used in clinical practise. Analysis showed that LASr mean was higher in male group without AF (*M* = 27.81; SD = 9.16) and lower in males with AF (*M* = 18.8; SD = 9.04); *U* = 105; *p* = 0.015. The second parameter, LASI, was higher in men with AF (*M* = 0.91; SD = 0.56) and lower in male population without AF (*M* = 0.43; SD = 0.33); *U* = 68; *p* = 0.010. The results are shown in Table S5 and Graph S1.

### 3.5 LASr in the Entire Population

**Figure 4:**
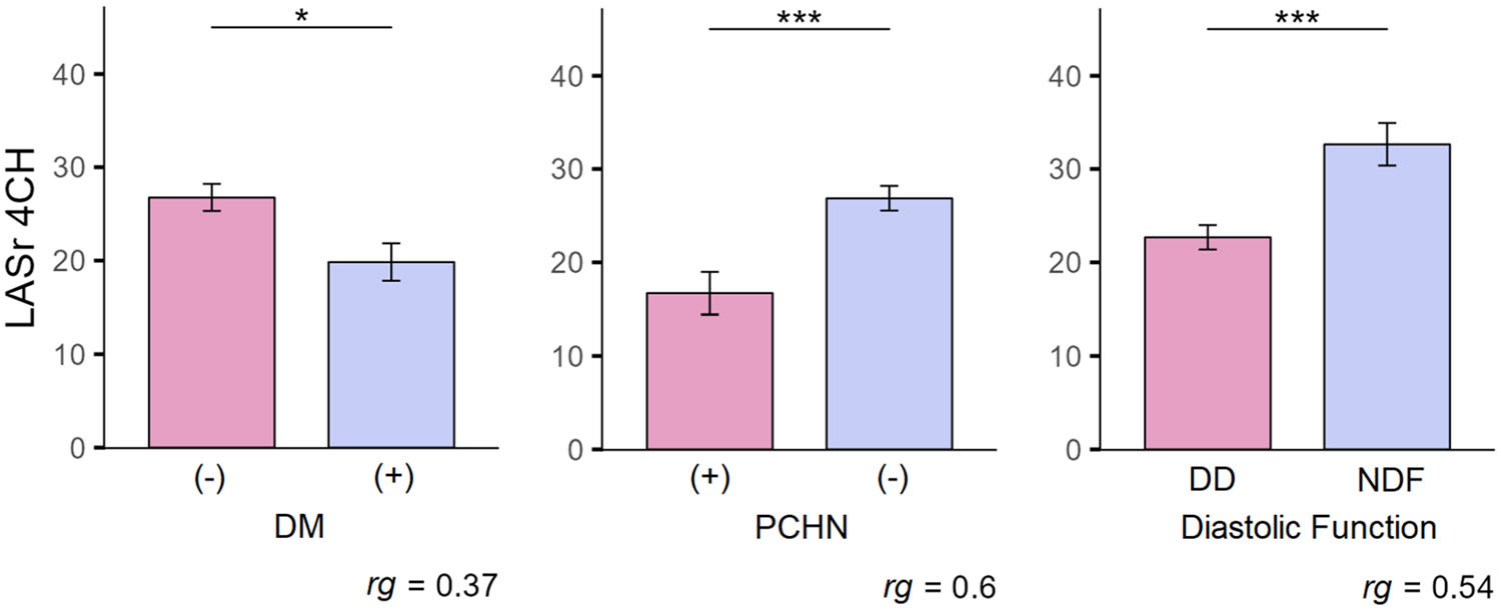
Comparison of LASr 4CH levels due to DM, CKD, Diastol. Note: Error whiskers represent the standard error of the mean. CKD, Chronic Kidney Disease; DD, Diastolic Dysfunction; DM, Diabetes Mellitus; NDF, Normal Diastolic Function; *rg*, Glass’ biserial correlation statistic; *p < 0.05. **p < 0.01. ***p < 0.001.

A separate analysis was performed of LASr 4CH in the entire study population. The analysis showed that higher LASr 4CH was in patients without DM (*M* = 26.76%; SD = 10.6) compared to those with DM (*M* = 19.83%; SD = 8.5); *U *= 305.5; *p* = 0.019. LASr 4CH was also higher in patients without chronic kidney disease, CKD(−),(*M* = 26.86%; SD = 10.1) compred to patients with kidney disease, CKD (+), (*M* = 16.69%; SD = 8.23); *U* = 152; *p* < 0.001. Higher LASr 4CH was noted in patients with normal left ventricle diastolic function, NDF (*M* = 32.65%; SD = 9.39) compared to those with diastolic dysfunction, DD (*M* = 22.74%; SD = 9.82); *U* = 209; *p*: 0.001. The results are shown in Table S6 and Figure 4.

### 3.6 Relationship Analysis

**Figure 5:**
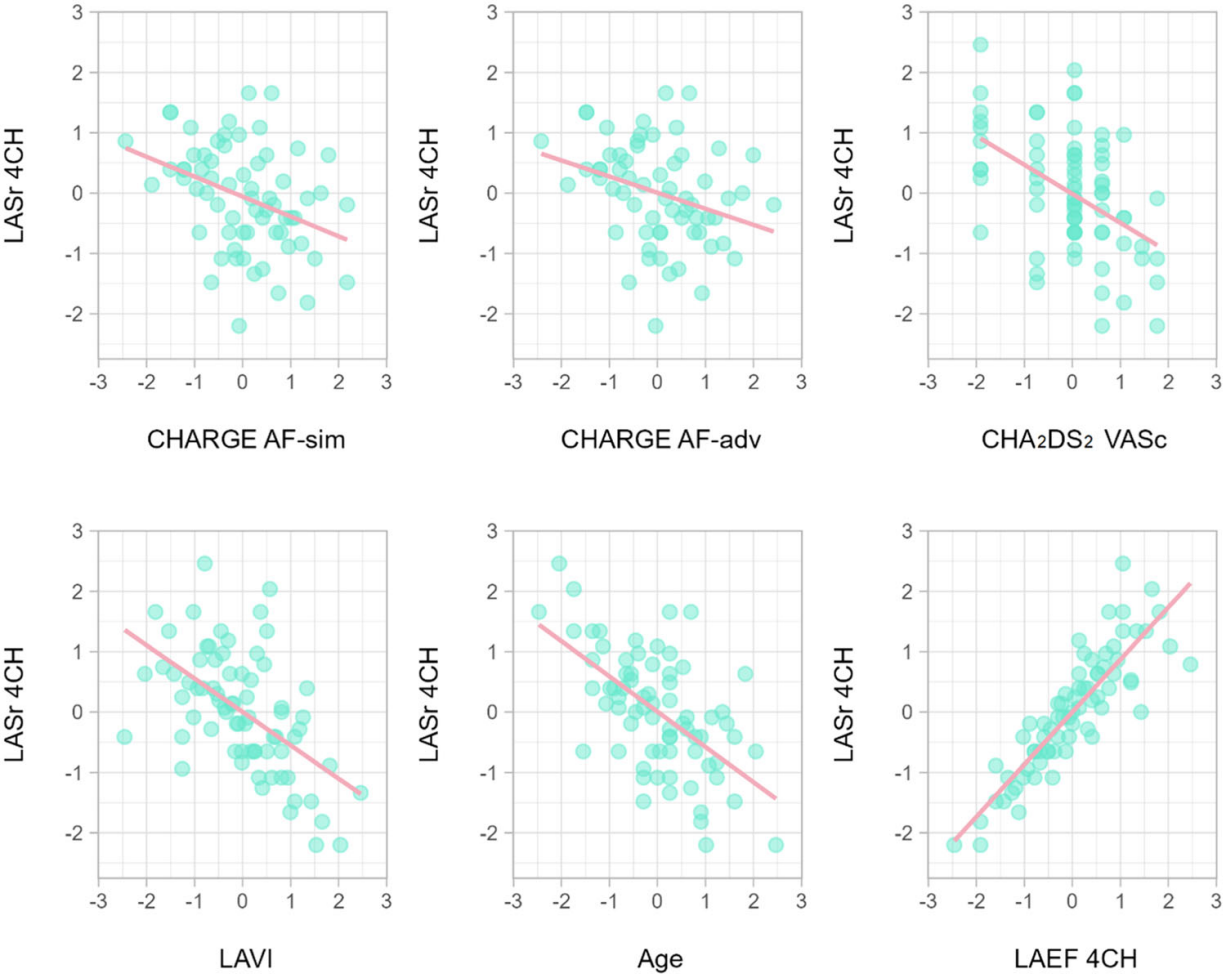
Relationships between CHARGE AF‐sim (basic model CHARGE AF calculator), CHARGE AF‐adv (extended model CHARGE AF calculator), CHA_2_DS_2_ VASc scale, LAVI (Left Atrium Volume Index), Age, LAEF4CH (total Left Atrium Ejection Fraction in apical four chamber view) and LASr 4CH (Left Atrial reservoir strain in four chamber view).

The analysis found that a decrease in LASr 4CH was significantly related to: (a) an increase in risk calculated using CHARGE AF sim and (b) using CHARGE AF adv, (c) an increase in risk using CHA_2_DS_2_ VASc, (d) an increase in LAVi, (e) an icrease in Age, (f) a decrease in LAEF 4CH. The results are shown in Figure 5.

## 4 Discussion

The study showed that echocardiographic parameters such as LASr, which means PALS also, LAEF, LAarea, LASI, LVEDd, LVGLS, PALS + LVGLS, E/e’ may be indicators of atrial fibrillation risk in patients with acute myocardial infarction.

AF was diagnosed in 5.4% of cases during the long‐term follow‐up period, which is comparable to results obtained in the first, sparse, reports confirming that reduced LASr values in patients with myocardial infarction indicate an increased risk of fututre AF [23]. In a population‐based study evaluating the incidence of AF among patients with myocardial infarction with a follow‐up period of 6.6 years, an arrhythmia that first occurred more than 30 days after myocardial infarction was diagnosed in 12% of the subjects [24]. In a study standing out among others due to its methodology, using an Implantable Cardiac Monitor (ICM) in patients after myocardial infarction, with reduced left ventricular ejection fraction, arrhythmia was first diagnosed in 39.3% of subjects. Not to be overlooked is the fact that the study population included a special profile of patients. Nevertheless, special attention should be given to the observation that more than 90% of the cases were asymptomatic, which may be influenced by the beta blocker used after myocardial infarction [25]. It is worth citing the REHEARSE‐AF study, which confirmed that when a single‐lead electrocardiogram was performed twice a week using a smartphone, AF was detected 3.9 times more often in patients aged 65 and older [26]. It is uncertain whether the differences in AF diagnosis rates are due to baseline characteristics of the population or to different methods of searching for arrhythmia. During the long‐term follow‐up of the main project presented here, 71% patients diagnosed with AF denied symptoms of arrhythmia. It cannot be excluded that a significant proportion of episodes went undetected due to insufficiently effective screening methods, especially in the situation of using a beta blocker recommended after myocardial infarction which, by affecting symptom reduction, contributes to a decrease in clinical alertness. Diagnosis of AF in a patient with myocardial infarction has a direct impact on the pharmacotherapy resulting from an increase in scores on the CHA_2_DS_2_ VASc that estimates the risk of thromboembolic complications. Both in the acute and long‐term phases, the above constellation changes pharmacotherapy significantly, also affecting the modification of antiplatelet theraphy. In summary, the implications of AF diagnosis are significant. Insufficiently effective screening methods delaying diagnosis may contribute to the development of atrial myopathy, which, at the histological level, is brought about by intercellular fibrosis, glycogen accumulation in atrial cardiomyocytes and progressive loss of sarcomeres [27]. This is most likely due to the complex interaction of various factors, such as endothelial dysfunction, inflammatory processes, atrial tensioning forces, the aging process, genetic and neurohumoral factors [28, 29]. It should be emphasized that atrial myopathy currently remians a pathophysiological concept rather than a clinical entity with specific management criteria. By design, the project presented used currently recommended, routine sreening methods. The diagnostic model reflected current clinical practice. Looking through the prism of the data cited and the results obtained, attempts could be made to modify the existing methods. Using echocardiography in determining the condition of the left atrium, and consequently constructing an AF risk profile, together with the use of modern electronic screening systems, a multidirectional and multifaceted approach can be attempted. It seems that a individual approach to the patient could help improve diagnostic efficiency. Given that patients with AF are a significant burden on the healthcare system, it seems obvious that improvement in detecting AF before the complication stage would contribute to a reduction in financial outlays.

## 5 Limitations

It should be noted that significant limitations were the size of the study group, the duration of the study, and the in‐hospital isolation of patients due to SARS‐CoV‐2 infection. An indirect consequence was the number of endpoints in long‐term follow‐up.

## 6 Conclusions

Echocardiography, despite having been known for several decades, is constantly being improved both in terms of methodology and parameters. Currently, in daily clinical practice, it is possible to use not only its classical forms but also advanced echocardiographic techniques, which can increase the efficiency of atrial fibrillation detection. The conducted research has shown that among the echocardiographic parameters there are some which can indicate the risk of atrial fibrillation. Taking into account the approachable methodology and the required time for additional measurements, the crucial parameters are LASr and LASI in determining which we use LASr also as well as E and e’. E/e’ is determined routinely while assesing the left ventricle diastolic function. Concluding, LASr possesses great potential to be implemented in daily clinical practice.

Characterizing and distinguishing a group of patients at particularly high risk for arrhythmia occurrence using the mentioned echocardiographic parameters namely LASr and LASI, with the subsequent modification of existing screening methods in this group of patients, could have a real impact on the frequency of arrhythmia diagnosis, reduction of complications and reduction of healthcare costs. Although the study group was not large, even on its basis it can be suspected that a large percentage of arrhythmias is not effectively diagnosed. As it seemed pointless to continue the study, collect data and extend the group, given using routine sreening methods, the recruitment was completed with 74 patients. The project presented may inspire further studies with larger group sizes, but they should be based on more effective sreening methods using new diagnostic tools.

## Author Contributions

**Beata Jacuś:** conceptualization, methodology, software, validation, formal analysis, investigation, data curation, writing – original draft preparation, visualization. **Anna Milewska:** software, validation, formal analysis, data curation, visualization. **Paweł Miękus:** writing – review and editing. **Marcin Konarzewski:** investigation. **Ludmiła Daniłowicz‐Szymanowicz:** conceptualization, methodology, writing – review and editing. **Andrzej Lubiński:** writing – review. **Grzegorz Grześk:** conceptualization, methodology, software, validation, writing – review and editing, supervision. All authors have read and agreed to the published version of the manuscript.

## Conflicts of Interest

The authors declare no conflicts of interest.

## Supporting information

Supporting information.

## Data Availability

The corresponding author has complete access to the original study data, and anonymized data will be made available upon request with a reasonable purpose.
