# Supplementary material for: Assessment of Risk Factors for Atrial Fibrillation With a Particular Focus on Echocardiographic Parameters, in Patients With Acute Myocardial Infarction
Source: Clin Cardiol. 2025 Mar 28;48(4):e70114. doi: 10.1002/clc.70114 (PMC11950838; doi:10.1002/clc.70114)
Supplement: Supplementary file 1 — Supporting information. [file CLC-48-e70114-s001.docx]

SUPPLEMENTARY

| Table S1. ***Baseline clinical characteristics of the population.*** | | | |
| --- | --- | --- | --- |
| Variables | AF(-) (*n* = 58) | AF(+) (*n* = 14) | *p* |
| Age [years] | 63.5 ± 11.39 | 71.79 ± 13.61 | 0.047 |
| Gender |  |  | 1.000 |
| Female | 15 (26) | 4 (29) |  |
| Male | 43 (74) | 10 (71) |  |
| Hospitalization (days) | 4.58 ± 1.18 | 5.5 ± 2.1 | 0.103 |
| STEMI/NSTEMI |  |  | 0.933 |
| NSTEMI | 24 (41) | 5 (36) |  |
| STEMI | 34 (59) | 9 (64) |  |
| BMI – Body Mass Index (kg/m^2^) | 26.76 ± 3.75 | 30.15 ± 5.32 | 0.039 |
| BSA (m^2^) | 1.93 ± 0.21 | 2.02 ± 0.19 | 0.178 |
| Systolic RR (mmHg) | 139.59 ± 25.33 | 130.11 ± 26.87 | 0.197 |
| Diastolic RR (mmHg) | 79.31 ± 12.33 | 79.79 ± 14.52 | 0.831 |
| MAP (mmHg) | 99.38 ± 14.76 | 96.96 ± 16.53 | 0.659 |
| CAD prior |  |  | 0.438 |
| No | 50 (86) | 11 (79) |  |
| Yes | 8 (14) | 3 (21) |  |
| MI prior |  |  | 1.000 |
| No | 51 (88) | 13 (93) |  |
| Yes | 7 (12) | 1 (7) |  |
| PCI prior |  |  | 0.366 |
| No | 51 (89) | 11 (79) |  |
| Yes | 6 (11) | 3 (21) |  |
| CABG prior |  |  | 0.095 |
| No | 57 (98) | 12 (86) |  |
| Yes | 1 (2) | 2 (14) |  |
| Stroke prior |  |  | 0.483 |
| No | 56 (97) | 13 (93) |  |
| Yes | 2 (3) | 1 (7) |  |
| Diabetes mellitus |  |  | 0.016 |
| No | 46 (79) | 6 (43) |  |
| Yes | 12 (21) | 8 (57) |  |
| CKD |  |  | 0.128 |
| No | 49 (84) | 9 (64) |  |
| Yes | 9 (16) | 5 (36) |  |
| Thyroid diseases |  |  | 0.679 |
| No | 48 (83) | 13 (93) |  |
| Yes | 10 (17) | 1 (7) |  |
| COVID-19 |  |  | 0.217 |
| No | 32 (56) | 11 (79) |  |
| Yes | 25 (44) | 3 (21) |  |
| COVID-19 vaccination |  |  | 0.719 |
| No | 13 (23) | 2 (14) |  |
| Yes | 44 (77) | 12 (86) |  |
| ECG LVH |  |  | 1.000 |
| No | 53 (91) | 13 (93) |  |
| Yes | 5 (9) | 1 (7) |  |
| CHARGE AF-sim (%) | 3.84 ± 3 | 5.26 ± 5.17 | 0.617 |
| CHARGE AF-adv (%) | 3.77 ± 2.99 | 3.96 ± 4.17 | 0.836 |
| CHA_2_DS_2_ VASc (points) | 3.02 ± 1.43 | 4.14 ± 1.75 | 0.041 |
| Beta blocker at discharge |  |  | 1.000 |
| No | 7 (12) | 2 (14) |  |
| Yes | 51 (88) | 12 (86) |  |
| ACE-I/ARB at discharge |  |  | 1.000 |
| No | 3 (5) | - |  |
| Yes | 55 (95) | 14 (100) |  |
| MRA at discharge |  |  | 0.231 |
| No | 50 (86) | 10 (71) |  |
| Yes | 8 (14) | 4 (29) |  |
| SGLT-2i at discharge |  |  | 0.648 |
| No | 52 (90) | 12 (86) |  |
| Yes | 6 (10) | 2 (14) |  |
| *Note:* values represent *mean ± SD, n* (%)  STEMI – ST-T Segment Elevation Myocardial Infarction; NSTEMI – non ST-T Segment Elevation Myocardial Infarction; BSA – Body Surface Area; MAP – Mean Arterial Pressure; CAD – Coronary Artery Disease; MI – Myocardial Infarction; PCI – Percutaneous Coronary Intervention; CABG – Coronary Artery Bypass Grafting; CKD – Chronic Kidney Disease; ECG LVH – ECG Left Ventricular Hypertrophy; ACE-I – angiotensin converting enzyme inhibitors; ARB – angiotensin 2 receptor blockers; MRA – aldosterone receptor antagonists; SGLT2i – sodium-glucose cotransporter-2 inhibitors; | | | |

| Table S 2**. *Baseline laboratory characteristics of the population.*** | | | |
| --- | --- | --- | --- |
| Variables | AF(-) (*n* = 58) | AF(+) (*n* = 14) | *p* |
| hsTroponin max (ng/ml) | 1.97 ± 2.23 | 2.68 ± 1.67 | 0.065 |
| CKMB max (ng/ml) | 57.22 ± 71.1 | 51.57 ± 94.91 | 0.660 |
| CRP max (mg/l) | 15.76 ± 25.07 | 26.32 ± 24.83 | 0.059 |
| Admission creatinine (mg/dl) | 0.91 ± 0.26 | 0.99 ± 0.36 | 0.589 |
| Maximum creatinine (mg/dl) | 0.98 ± 0.31 | 1.05 ± 0.37 | 0.477 |
| Admission eGFR (ml/min/1.^73m2^) | 58.51 ± 6.64 | 56.24 ± 7.86 | 0.146 |
| Minimum eGFR (ml/min.1.^73m2^) | 57.88 ± 7.78 | 55.25 ± 8.65 | 0.184 |
| TSH (mlU/l) | 2.29 ± 1.62 | 1.67 ± 1.12 | 0.278 |
| fT3 (pmol/l) | 4.08 ± 0.82 | 2.95 ± 1.13 | 0.155 |
| fT4 (pmol/l) | 15.43 ± 2.04 | 15.52 ± 2.05 | 0.836 |
| NTproBNP |  |  | 0.600 |
| Not determined | 53 (91) | 12 (86) |  |
| Normal | 1 (2) | - |  |
| Above normal | 4 (7) | 2 (14) |  |
| Total Cholesterol (mg/dl) | 176.48 ± 43.2 | 153 ± 46.68 | 0.034 |
| Triglycerides (mg/dl) | 153.9 ± 94.74 | 160.18 ± 78.96 | 0.623 |
| LDL – LDL Cholesterol (mg/dl) | 99.68 ± 32.1 | 81.5 ± 44.11 | 0.038 |
| HDL Cholesterol (mg/dl) | 44.91 ± 13.77 | 39.64 ± 8.77 | 0.172 |
| Admission Hb (g/dl) | 14.13 ± 1.79 | 13.89 ± 1.74 | 0.776 |
| Minimum Hb (g/dl) | 13.22 ± 1.49 | 12.38 ± 1.79 | 0.099 |
| Admission WBC (G/l) | 9.88 ± 3.02 | 10.58 ± 3.33 | 0.378 |
| Maximum WBC (G/l) | 10.52 ± 3.01 | 10.55 ± 2.71 | 0.909 |
| Admission neutrophils [%]. | 68.05 ± 11.86 | 73.24 ± 10.63 | 0.114 |
| Maximum neutrophils (%) | 70.26 ± 10.53 | 76.94 ± 8.82 | 0.023 |
| Admission lymphocytes (G/l) | 22.22 ± 9.89 | 18.04 ± 9.12 | 0.126 |
| Maximum lymphocytes (G/l) | 27.46 ± 8.36 | 27.76 ± 9.6 | 0.803 |
| Admission platelet count (G/l) | 238.22 ± 69.41 | 208.92 ± 54.8 | 0.161 |
| Minimum platelet count (G/l) | 214.33 ± 62.3 | 173.78 ± 51.13 | 0.050 |
| Electrolyte imbalance |  |  | 0.708 |
| No | 48 (83) | 11 (79) |  |
| Yes | 10 (17) | 3 (21) |  |
| *Note:* values represent *mean ± SD, n* (%)  CRP – C-reactive protein; eGFR – estimated Glomerular Filtration Rate (using MDRD equation); Hb – hemoglobin; WBC – white blood cells; | | | |

| Table S 3. ***Baseline catheter laboratory characteristics of the population.*** | | | |
| --- | --- | --- | --- |
| Variables | AF(-) (*n* = 58) | AF(+) (*n* = 14) | *p* |
| Coronarography-performance time |  |  | 0.679 |
| One day | 48 (83) | 13 (93) |  |
| More than 24 hours | 10 (17) | 1 (7) |  |
| Time until coronarography |  |  | 0.970 |
| ≤2 h | 4 (7) | 1 (7) |  |
| 2-6 h | 15 (26) | 3 (21) |  |
| 6-12 h | 10 (17) | 2 (14) |  |
| 12-24 h | 10 (17) | 2 (14) |  |
| >24 h | 19 (33) | 6 (44) |  |
| MVCAD |  |  | 0.596 |
| No | 32 (55) | 6 (43) |  |
| Yes | 26 (45) | 8 (57) |  |
| Type of treatment |  |  | 1.000 |
| Invasive | 55 (95) | 14 (100) |  |
| Non-invasive | 3 (5) | - |  |
| Until the next PCI |  |  | 0.645 |
| No | 35 (60) | 10 (71) |  |
| Yes | 23 (40) | 4 (29) |  |
| Until CABG |  |  | 1.000 |
| No | 55 (95) | 13 (93) |  |
| Yes | 3 (5) | 1 (7) |  |
| *Note:* values represent *mean ± SD, n* (%)  MVCAD – Multivessel Coronary Aretry Disease; PCI – Percutaneous Coronary Intervention; CABG – Coronary Artery Bypass Grafting; | | | |

| Table S 4. ***Baseline echocardiographic characteristics of the population.*** | | | |
| --- | --- | --- | --- |
| Variables | AF(-) (*n* = 58) | AF(+) (*n* = 14) | *p* |
| LAS FPS | 68.67 ± 9.08 | 65.92 ± 6.01 | 0.579 |
| LA PLAX (mm) | 39.8 ± 5.16 | 39.93 ± 2.91 | 0.569 |
| LA area (cm^2)^ | 18.84 ± 3.59 | 21.62 ± 4.2 | 0.027 |
| LAVI 4CH AFI (ml/m^2^) | 25.34 ± 9.11 | 29.69 ± 9.43 | 0.123 |
| LAVI bip AFI (ml/m^2^) | 25.72 ± 8.25 | 31.07 ± 10.5 | 0.109 |
| LAVI 4CH disc (^ml/m2^) | 27.68 ± 7.35 | 30.88 ± 8.26 | 0.201 |
| LAVI bip disc (ml/m^2^) | 27.45 ± 7.45 | 32.06 ± 10.59 | 0.091 |
| LAEF 4CH (%) | 55.93 ± 14.29 | 43.46 ± 20.22 | 0.029 |
| LAEF mean (%) | 55.63 ± 12.52 | 44.08 ± 16.58 | 0.014 |
| EI 4 CH | 1.51 ± 0.92 | 1.14 ± 1.05 | 0.068 |
| EI | 1.49 ± 0.76 | 1.03 ± 0.73 | 0.032 |
| passive EF (cd) 4CH (%) | 24.7 ± 14.55 | 23.11 ± 11.69 | 0.874 |
| passive EF (cd) mean (%) | 23.28 ± 12.53 | 21.76 ± 7.44 | 0.976 |
| active EF (pump) 4CH (%) | 41.44 ± 13.88 | 36.56 ± 18.94 | 0.432 |
| active EF (pump) mean (%) | 41.25 ± 12.18 | 37.56 ± 14.66 | 0.562 |
| LASr 4CH (%) | 26.72 ± 9.77 | 19.08 ± 11.42 | 0.020 |
| LASr mean (%) | 26.73 ± 9.54 | 18.62 ± 8.79 | 0.009 |
| LAS cd 4CH (%) | -12.68 ± 6.58 | -9.54 ± 4.65 | 0.089 |
| LAS cd mean (%) | -12.74 ± 6.71 | -9.75 ± 2.73 | 0.147 |
| LAS ct 4CH (%) | -13.7 ± 6.1 | -12.89 ± 6.97 | 0.764 |
| LAS ct mean (%) | -13.91 ± 6.05 | -12.11 ± 5.34 | 0.555 |
| LAFI | 58.37 ± 26.68 | 43.38 ± 37.81 | 0.057 |
| E wave (m/s) | 0.72 ± 0.19 | 0.83 ± 0.18 | 0.041 |
| E/e' | 9.58 ± 3.51 | 12.62 ± 3.76 | 0.010 |
| LASI | 0.45 ± 0.35 | 0.95 ± 0.66 | 0.016 |
| LVMI (g/m^2^) | 100.09 ± 19.99 | 107.77 ± 19.67 | 0.207 |
| LVEDd (mm) | 49.79 ± 5.22 | 52.92 ± 3.73 | 0.022 |
| RWT | 0.41 ± 0.05 | 0.39 ± 0.04 | 0.633 |
| IVS (mm) | 10.88 ± 0.98 | 11 ± 1.08 | 0.599 |
| PW (mm) | 10.09 ± 1.17 | 10.38 ± 1.12 | 0.247 |
| LV GLS (%) | -14.49 ± 3.92 | -11.47 ± 4.2 | 0.021 |
| PALS+LV GLS (%) | 39.22 ± 13.95 | 27.11 ± 12.79 | 0.005 |
| *Note:* values represent *mean ± SD, n* (%)  LAS FPS – Left Atrial Strain Frames per Second; LA PLAX – Left Atrium Parasternal Long Axis View; LA area – area of the left atrium; LAVi – Left Atrium Volume Index; LAEF – Left Atrium Ejection Fraction (total); EI – Expansion Index; LASr – Left Atrial Strain (reservoir); LAScd – Left Atrial Strain (conduit); LASct – Left Atrial Strain (contraction); LAFI – Left Atrium Functional Index; LASI – Left Atrium Stiffness Index; LVMI – Left Ventricle Mass Index; LVEDd – Left Ventricle End Diastolic diameter; RWT – Relative Wall Thickness; IVS –Interventricular Septum; PW –Posterior Wall; LV GLS – Left Ventricle Global Longitudinal Strain; PALS – Peak Atrial Longitudinal Strain;  Table S5   \| Table S 5. *Differences between groups distinguished based on the Arrhythmia variable in terms of the level of the LASr mean and LASI variables (Male Group)* \| \| \| \| \| --- \| --- \| --- \| --- \| \| Variables \| AF (-) (*n* = 43) \| AF (+) (*n* = 10) \| *p* \| \| LASr mean [%]. \| 27.81 ± 9.16 \| 18.8 ± 9.04 \| 0.015 \| \| LASI \| 0.43 ± 0.33 \| 0.91 ± 0.56 \| 0.010 \| \| *Note*: values represent *mean ± SD* \| \| \| \|   Table S6   \| Table S6. *Differences in LASr 4CH levels depending on DM, CKD and Diastolic function* \| \| \| \| \| --- \| --- \| --- \| --- \| \|  \| DM (-) (*n* = 54) \| DM (+) (*n* = 18) \| *p* \| \| LASr 4CH [%]. \| 26.76 ± 10.6 \| 19.83 ± 8.5 \| 0.019 \| \|  \| CKD (-) (*n* = 59) \| CKD (+) (*n* = 13) \|  \| \| LASr 4CH [%]. \| 26.86 ± 10.1 \| 16.69 ± 8.23 \| < 0.001 \| \|  \| DD (*n* = 55) \| NDF (*n* = 17) \|  \| \| LASr 4CH [%]. \| 22.74 ± 9.82 \| 32.65 ± 9.39 \| < 0.001 \| \| *Note*: values represent *mean ± SD;* DD = Diastolic Dysfunction; NDF = Normal Diastolic Function; CKD= Chronic Kidney Disease; LASr4CH- Left Atrial reservoir Strain in apical four chamber view; \| \| \| \|   GRAPH S1  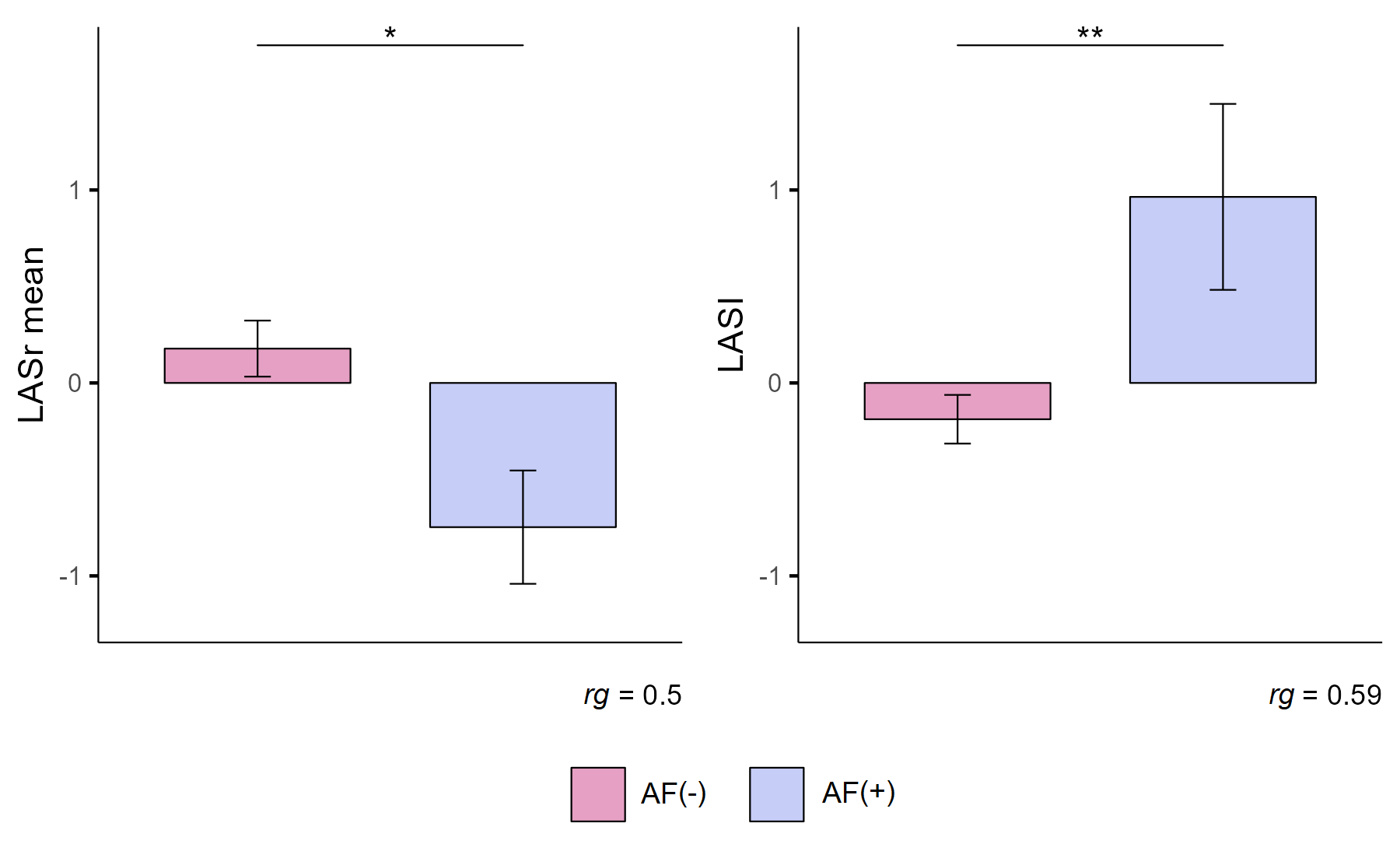  Graph S1. *Comparison of groups of patients with a diagnosis of arrhythmia (atrial fibrillation) and without arrhythmia in terms of variables: LASr mean (Left Atrial reservoir Strain, mean value), LASI (Left Atrium Stiffness Index, Male Group).*  *Note*: Results are expressed in the standardized unit of measurement; Error whiskers represent the standard error of the mean. *rg* = Glass' biserial correlation statistic;  * . p < .05. ** . p < .01. *** . p < .001. | | | |
